# Occurrence of Circulating Antibodies against the Hemagglutinins of Influenza Viruses in the 2022/2023 Epidemic Season in Poland

**DOI:** 10.3390/v16071105

**Published:** 2024-07-09

**Authors:** Katarzyna Kondratiuk, Anna Poznańska, Karol Szymański, Emilia Czajkowska, Bartosz Mańkowski, Lidia B. Brydak

**Affiliations:** 1 Laboratory of Influenza Viruses and Respiratory Viruses, Department of Virology, National Institute of Public Health NIH—National Research Institute, 00-791 Warsaw, Poland; kszymanski@pzh.gov.pl (K.S.); eczajkowska@pzh.gov.pl (E.C.); bmankowski@pzh.gov.pl (B.M.); lbrydak@pzh.gov.pl (L.B.B.); 2 Department of Population Health Monitoring and Analysis, National Institute of Public Health NIH—National Research Institute, 00-791 Warsaw, Poland; paula@pzh.gov.pl

**Keywords:** influenza, hemagglutinin antibodies, GMT, protection rate, serum, antibody, RNA viruses

## Abstract

The aim of this study was to determine the level of anti-hemagglutinin antibodies in blood sera collected from patients during the 2022/2023 epidemic season in Poland. A total of 700 sera samples from patients across the country were tested. The samples were divided into seven groups according to the age of the patients, with 100 samples from each age group. The hemagglutination inhibition test (OZHA) was used to determine the level of anti-hemagglutinin antibodies. The test results have confirmed the presence of anti-hemagglutinin antibodies for antigens A/Victoria/2570/2019 (H1N1)pdm09, A/Darwin/9/2021 (H3N2), B/Austria/1359417/2021 (B/Yamagata lineage) and B/ Phuket/3073/2013 (B/Victoria lineage) present in the influenza vaccine recommended by the World Health Organization (WHO) for the 2022/2023 epidemic season. The highest geometric mean antibody titres (GMT) and protection rate values (%) were recorded for hemagglutinin A/H3N2. In Poland, in the 2022/2023 epidemic season, the percentage of the population vaccinated against influenza was 5.7%. Therefore, the test results can be interpreted as the response of the immune system in patients who have been previously infected with an influenza virus.

## 1. Introduction

Influenza is an acute infectious disease of the respiratory system caused by influenza viruses, occurring every season and presenting as an acute respiratory infection. The two types responsible for the usual seasonal increases in influenza cases are influenza A and B viruses. What distinguishes influenza from the common cold is not only its rapid course, but also complications that are dangerous to health and can be life-threatening. Seasonal influenza is considered a major health problem and a significant economic burden for public health care [1].

Influenza is a threat to all age groups, especially the elderly, children and people with comorbidities, leading to a significant increase in the mortality rate among people from these groups. People over 65 years of age as well as patients with comorbidities such as diabetes or other chronic diseases (including asthma, chronic kidney disease, chronic heart failure, and immunosuppression) are considered particularly susceptible to severe influenza infection [2,3].

The advent of the COVID-19 pandemic, caused by a new type of SARS-CoV-2 virus, had a significant impact on the epidemiology of other infectious diseases, including influenza. This was a unique epidemic season, when the reported incidence of influenza remained at a very low level in many countries. Stringent restrictions and various measures introduced in March 2020 to limit the spread of the virus contributed to a sudden decline in the incidence of influenza and other respiratory viruses. Another reason could be the natural viral competition resulting from the fact that one virus usually dominates in the population at a given time (here, the SARS-CoV-2 virus) [2,4,5]. However, already in the 2022/2023 epidemic season in Europe, a four-fold increase in influenza cases was recorded compared to the previous seasons [5]. A limited spread of influenza viruses in the population may contribute to a reduction in herd immunity and increase the population’s susceptibility to possible future outbreaks [4]. This only emphasizes the importance of influenza vaccination in the prevention of influenza, especially in the context of protecting people in high-risk groups against the potential risk of serious post-influenza complications and preventing future epidemics.

The World Health Organization (WHO) estimates that approximately 3–5 million patients suffer from severe influenza symptoms each year worldwide [6].

Based on mathematical models, it is estimated that influenza-related mortality rates typically range from 4.0 to 8.8 per 100,000 population worldwide (per 100,000 individuals) and from 51.3 to 99.4 per 100,000 population aged 75 and older [7].

Vaccinations are the cheapest and the most effective method of prevention. The administration of the vaccine induces an immune response, which is the production of antibodies directed against the influenza virus antigens contained in the vaccine. In Poland, in the 2022/2023 epidemic season, quadrivalent vaccines that provided active immunity against four strains of influenza virus were available: against two subtypes of type A influenza—A/H1N1/pdm09 and A/H3N2/—and against type B influenza of B/Victoria and B/Yamagata lineages. The quadrivalent vaccine increases the level of vaccination effectiveness. Due to the frequency of mutations of influenza viruses, it is necessary to update the composition of the available vaccines every season. The recommended composition of influenza vaccines is established on the basis of the data collected by 152 National Influenza Centres (NIC) in laboratories around the world operating under the patronage of WHO. In Poland, NIC operates at the National Institute of Public Health—National Research Institute.

Vaccination decreases the incidence of influenza by 40–70%, depending on the flu season and the population of people vaccinated. The duration of post-vaccination immunity to infection with virus strains included in the vaccine or even related strains may vary, but usually ranges from 6 to 12 months. This is why seasonal flu vaccinations are so important [8]. 

The most important viral antigens with a leading role in the pathophysiology of the infection are hemagglutinin and neuraminidase.

Neuraminidase and hemagglutinin facilitate the attachment of the influenza virus particle to the surface of the infected cell. These are antigens that stimulate the formation of specific neutralizing antibodies. 

It is assumed that high titres of antibodies against hemagglutinin (above ≥1:40) can ensure a high level of protection against the infection caused by influenza viruses [9]. They mainly belong to the IgG class. Antibodies that inhibit neuraminidase have been shown to contribute to the body’s resistance but do not prevent influenza infections even at high titres in blood serum. However, they block the replication process, reduce the incidence of the disease and alleviate the course of the infection if and when it occurs [9,10,11].

The aim of this study was to determine the level of anti-hemagglutinin antibodies of influenza viruses in the sera collected from patients in different age groups during the 2022/2023 epidemic season in Poland.

## 2. Materials and Methods

In this study, 700 blood serum samples were collected from patients by technicians at 16 Provincial Sanitary and Epidemiological Stations in Poland, in accordance with the recommendations of the World Health Organization (WHO), between 1 October 2022 and 31 September 2023, i.e., during the 2022/2023 influenza epidemic season. Serum samples were collected from patients hospitalized due to a respiratory infection. The sera were divided into 7 groups depending on the age of the patients: 0–4, 5–9, 10–14, 15–25, 26–44, 45–64 and ≥65 years of age. The study material consisted of 100 serum samples from each of the above age groups (the number of samples collected in each province was different). The samples were stored at −80 °C before the tests.

All the viruses used in this study were obtained from the Worldwide Influenza Centre at the Crick Institute, London, working in close collaboration within the WHO Global Influenza Surveillance Network. They were then multiplied at the National Influenza Centre, National Institute of Public Health—National Research Institute (NIZP PZH-PIB), according to WHO recommendations [10]. The method of multiplying 11-day-old chicken embryos in the allantois was used. After inoculation with the appropriate virus, chicken embryos were incubated under the following conditions: for A/H3N2/ and A/H1N1/pdm09 viruses, 2 days at 37 °C; and for influenza B viruses, 3 days at 35 °C. After determining the titre of each influenza virus propagated, the appropriately labelled vials containing the viruses were stored at −80 °C until tests were conducted [10]. This study used antigens for the 2022/2023 epidemic season recommended by WHO (Table 1).

The level of anti-hemagglutinin antibodies was determined by a hemagglutination inhibition test (OZHA). This assay is normally used to determine the level of specific anti-influenza antibodies, and for this study, 8 viral hemagglutination units were used. Before the assay, the sera were inactivated according to the accepted standards [10,12]. A 1:8 solution of each of the four viruses used in the test was prepared. Alsever’s solution and PBS buffer used for the determination of anti-hemagglutinin antibody levels are prepared in-house. In this study, colourless and transparent microtitre plates with a V-shaped bottom were used. The OZHA test uses turkey red blood cells (RBC) suspended in Alsever’s solution and delivered to the laboratory. The solution acts as an anticoagulant and blood preservative. The red blood cell concentrate necessary for the test was obtained by centrifugation at 1200 rpm for 10 min. In order to obtain the appropriate blood cell concentration for the HAI test, the following proportion was used: 999 µL PBS: 5 µL packed cells (concentration of RBC: 0.5%). Before testing, each serum was treated with a receptor-destroying enzyme (RDE) (SIGMA-ALDRICH, St. Louis, MO, USA) at 37 °C for 16 h. The serum–RDE mixture was then incubated at 56 °C for 30 min to inactivate the enzyme. In the OZHA assay, serial dilutions of the sera were made in PBS buffer on a plate (25 µL in every row). To the first row, 50 µL of the patient’s prepared serum was pipetted, and then, a serial dilution was made in PBS. Then, the prepared virus solution with a titre of 1:8 was added to the plate with diluted viruses and incubated for 15 min at room temperature. After the 15 min incubation, 50 µL of blood cell solution was added and incubated for 30 min at room temperature. The results were then read. The plate was lifted and tilted to allow RBCs to run down to the side of the well. If an antigen–antibody reaction occurred, haemagglutination of the RBCs was inhibited. The existence of haemagglutination indicates that the antigen is homologous to the specific antibody that has been added. For each isolate, the row which presents no haemagglutination was observed; the specific antiserum that had been added in the wells of this row indicates the influenza type of the solute. The highest serum dilution which inhibits haemagglutination was recorded; this is the titre. The titres of hemagglutinin antibodies after a series of dilutions were as follows: 10, 20, 40, 80, 160, 320, etc. [10].

The results were later analysed based on the following parameters: the frequency of occurrence of individual anti-hemagglutinin antibodies in the tested sera according to age, the geometric mean antibody titre (GMT) of antibodies and the protection rate (%) (the percentage of people with anti-hemagglutinin antibodies at the level ≥1:40 achieved after the administration of the influenza vaccine or during past influenza infection) [8,10,13]. This value of the anti-hemagglutinin antibody titre is considered to be protective.

## 3. Statistical Analysis

All of the analysed values were determined along with the limits of a 95% confidence interval. The Chi-squared test was used to compare the age groups regarding the categorical variables (prevalence of anti-hemagglutinin antibodies and reaching their protective titre). The Kruskal–Wallis test was applied to compare the titre distribution between seven age groups, and the Mann–Whitney U test was used for two. For all the tests, the significance level was 0.05. The calculations were executed with the SPSS 12.0 PL.

## 4. Results

This study showed that among the study group, 242 patients had antibodies against A/Victoria/2570/2019 (H1N1)pdm09 (34.6% [31.1–38.1]) (the limits of the 95% confidence interval in square brackets). The largest group, i.e., the group with 566 patients (80.9% [78.0–83.8]), had antibodies against A/Darwin/9/2021 (H3N2) in their serum. Only 152 patients (21.7% [18.6–24.8]) had antibodies against B/Austria/1359417/2021 (B/Victoria lineage), while the antibodies against B/Phuket/3073/2013 (B/Yamagata lineage) were present in the sera of 442 patients (63.1% [59.5–66.7]).

**Figure 1:**
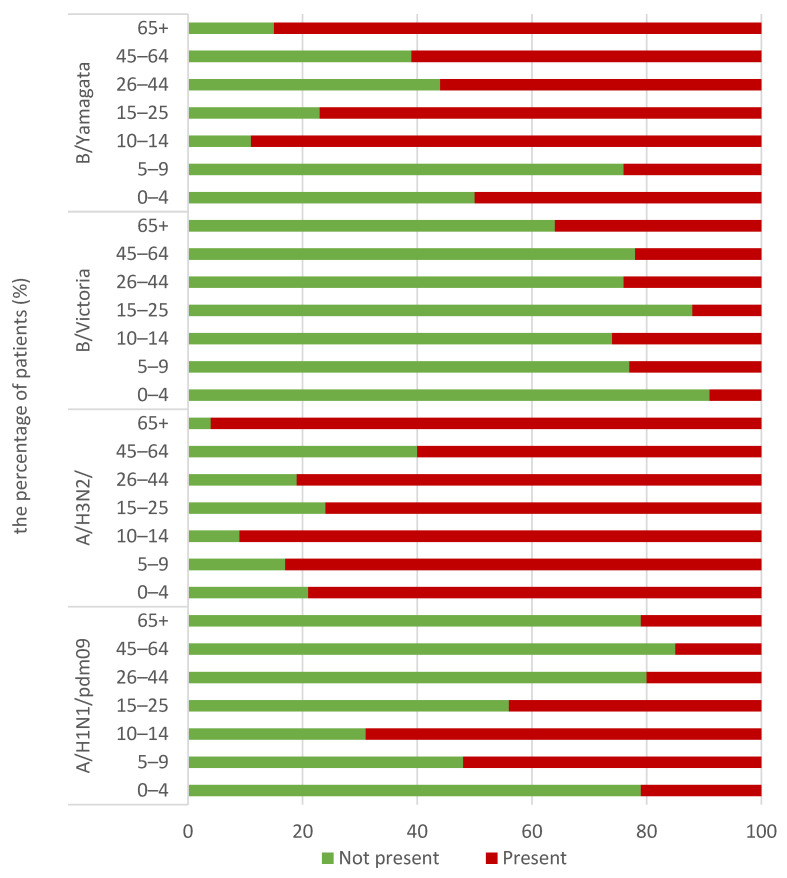
The presence of antibodies in the sera of patients aged 0–4, 5–9, 10–14, 15–25, 26–44, 45–64 and 65+ in the 2022/2023 epidemic season.

Figure 1 shows the percentage of patients in particular age groups (children and adults) who had antibodies against a particular influenza virus in the 2022/2023 epidemic season.

Statistically, the prevalence of each of the analysed antibodies in particular age groups varied significantly (in all cases *p* < 0.001). For antibodies against A/Victoria/2570/2019 (H1N1)pdm09, it ranged from 15 (8–22)% in the 45–64 age group to 69 (60–78)% in the 10–14 age group. The prevalence of antibodies against A/Darwin/9/2021 (H3N2) ranged from 60 (50–70)% in patients aged 45–64 years to 96 (92–100)% in the oldest patients aged 65+. The prevalence of antibodies against B/Austria/1359417/2021 (B/Victoria lineage) ranged from 9 (3–15)% in the group of the youngest children aged 0–4 to 36 (27–45)% in the group of the oldest people aged 65+. The prevalence of antibodies against B/Phuket/3073/2013 (B/Yamagata lineage) ranged from 24 (16–32)% in patients aged 5–9 years to 89 (83–95)% in patients aged 10–14.

The highest average antibody titre (191.6 [174.1–210.8]) was found for the A/Darwin/9/2021 (H3N2) subtype, whereas the values for other viruses were similar and amounted to 38.8 [33.4–45.0] for A/Victoria/2570/2019 (H1N1) pdm09, 35.5 [29.8–42.4] for B/Austria/1359417/2021 (B/Victoria lineage) and 34.0 [31.2–37.1] for B/Phuket/3073/2013 (B/Yamagata lineage).

**Figure 2:**
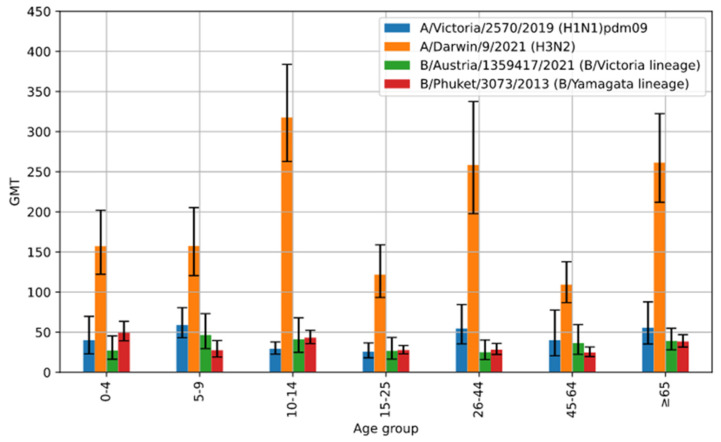
Geometric mean with 95% confidence intervals for titres of anti-hemagglutinin antibodies in patient sera according to age in the 2022/2023 epidemic season in Poland.

Figure 2 shows the geometric mean titres of anti-hemagglutinin antibodies (GMT) in the sera of patients in the particular age groups in the 2022/2023 epidemic season in Poland.

Statistically, the average level of antibodies against A/Victoria/2570/2019 (H1N1)pdm09, A/Darwin/9/2021 (H3N2) and B/Phuket/3073/2013 (B/Yamagata lineage) in the particular age groups varied significantly (*p* < 0.001), and only the average level of antibodies against B/Austria/1359417/2021 (B/Victoria lineage) in the particular age groups did not vary significantly. This difference is due to the fact that the latter antibodies were the least common (the compared groups were small; when calculating the geometric mean, only people with non-zero levels of antibodies were taken into account).

The highest GMT value for hemagglutinin A/H1N1/pdm09 was found in the sera of patients in the age group of 5–9 years (58.9 [43.1–80.5]), while the lowest GMT value was observed in the patients aged 15–25 years (25.7 [18.1–36.6]).

In the case of hemagglutinin A/H3N2/, the highest values were found in the sera collected from patients aged 10–14 years (317.6 [262.8–383.8]), and slightly lower values were recorded for patients in the age group of over 65 (261.4 [211.9–322.5]) and 26–44 years. (258.4 [197.7–337.6]). In the remaining age groups, GMT values for hemagglutinin A/H3N2/ were similar. The lowest value of 109.3 [86.7–137.8] was recorded for the age group of 45–64 years.

GMT values for influenza B antigens had lower values compared to influenza A. In the case of type B/Austria/1359417/2021, the highest GMT value was observed in the age group of 5–9 years (46.5 [29.6–73.1]), while the lowest GMT value was reported in patients aged 26–44 years (25.2 [15.8–40.2]). The highest GMT value for type B/Phuket/3073/2013 was found in the sera of the youngest patients aged 0–4 (49.9 [39.3–63.5]) and the lowest value in patients in the age group of 45–64 years (24.8 [19.5–31.6]).

**Figure 3:**
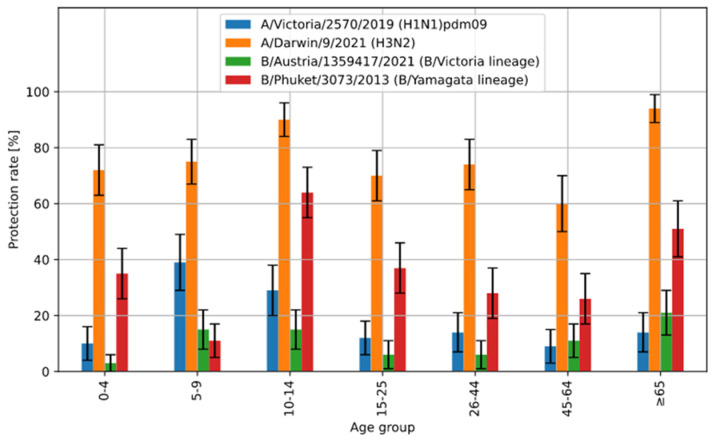
Percentage of cases with protective anti-hemagglutinin antibody titre (%), i.e., ≥40, with 95% confidence intervals in the 2022–2023 epidemic season, by age.

Figure 3 shows the percentage of cases with protective anti-hemagglutinin antibody titre (%), i.e., ≥40, in the 2022–2023 epidemic season, in various age groups. Statistically, the protective coefficients of antibody titres against all four influenza virus antigens in particular age groups varied significantly (*p* < 0.001).

Compared to the results of the protection rate for all antigens obtained in this study, the highest values of the protection rate were recorded for hemagglutinin A/H3N2/—76.4 [73.3–79.5]% for all patients. Relatively high values of the protection rate were also recorded for B/Yamagata line (B/Phuket): 36.0 [32.4–39.6]% (total number of patients). For A/H1N1/pdm09, this value was 18.1 [15.2–21.0]%, and for B/Austria/1359417/2021, it was 11.0 [8.7–13.3]%.

Antibodies against A/Victoria/2570/2019 (H1N1)pdm09 were statistically significantly more frequent (*p* < 0.001) in children up to 14 years of age (47.3 [417–52.9]%) than in the older population, i.e., aged 15 years and older (25.0 [20.8–29.2]%). Also, the antibody level of 40 or more was statistically significantly more prevalent in children (*p* < 0.001) (protective coefficients 26.0 [21.0–31.0]% vs. 12.3 [9.1–15.5]%). However, the average level of antibodies (GMT) in both groups was similar, as shown in Table 2.

Antibodies against A/Darwin/9/2021 (H3N2) were also statistically significantly more prevalent (*p* = 0.043) in children up to 14 years of age than in the older population (84.3 [80.2–88.4]% vs. 78.3 [74.3–82.3]%). However, neither the average level of antibodies (GMT) nor the value of the protective rate in both groups varied statistically. It is worth mentioning that in the 2021/2022 epidemic season, the protective levels for hemagglutinins H1 and H3 were more often observed in children aged 0–14 [13].

Statistically, the parameters for antibodies against B/Austria/1359417/2021 (B/Victoria lineage) did not vary significantly in the groups of children and the older population. Antibodies against B/Phuket/3073/2013 (B/Yamagata lineage) were statistically significantly more prevalent (*p* < 0.001) in people aged 15+ (69.8% [65.3–74.3] vs. 54.3% [48.7–59.9] in children). However, the average level of antibodies, if present, was statistically significantly (*p* < 0.001) higher in children (GMT = 42.3 vs. 30.0). As a result, the protection coefficient in both groups was similar (Table 2).

## 5. Discussion

This study confirmed the presence of antibodies in blood sera collected from patients during the 2022/2023 epidemic season in Poland. The antibodies to all four tested viruses (A/Victoria/2570/2019 (H1N1)pdm09, A/Darwin/9/2021 (H3N2), B/Austria/1359417/2021, and B/Phuket/3073/2013) were detected in patients of seven age groups. The percentage of cases with the protective anti-hemagglutinin antibody titre ≥40 in the 2022/2023 epidemic season varied among age groups and depended on the type of the influenza virus. In the 2022/2023 epidemic season, the highest percentage of cases with the protective anti-hemagglutinin antibody titre was observed for influenza A virus subtype A/H3N2/: A/Darwin/9/2021. A similar situation took place in the previous epidemic season 2021/2022, when the highest values of the protective coefficient were obtained for hemagglutinin H3 [13]. Due to the continuous circulation of respiratory viruses in the general population, it is necessary to introduce effective and quick diagnostic solutions, to ensure that appropriate treatment can be provided as quickly as possible, not only for the benefit of the patients but also for economic reasons [14]. The detection of neuraminidase-inhibiting antibodies in parallel with anti-hemagglutinin antibodies may help develop research on the immunogenicity and duration of antibody response to influenza vaccines [4].

The requirements of the Commission of the European Communities and the Committee for Proprietary Medicinal Products for influenza vaccination recommend achieving a protection rate of ≥70% among people aged 18–60 and ≥60% among older people [15,16]. In this study, the analyses show that protection rate values above 70% for hemagglutinin A/H3N2/ were achieved in the age groups 0–4, 5–9, 10–14, 15–25, 26–44 and 65+. In the age group of the oldest patients 65+, this value was the highest compared to all antigens and amounted to 94 [89–99]%. Protection rate values above 60% were only achieved for the B/Yamagata (B/Phuket) line in the age group of 10–14 years (64 [55–73]%. These data could correspond to a low level of vaccination against influenza during the epidemic season 2022–2023 in Poland (according to data from the Influenza Research Institute, National Influenza Centre). A high antibody titre may have been the result of a past infection caused by influenza viruses. At the same time, the percentage of the vaccinated population in the epidemic season 2022/2023 was only 5.7%. 

Recent clinical trials have shown that the influenza vaccine may have more beneficial properties than was expected and may even affect the course of COVID-19 infection. One study showed that vaccination against influenza was associated with a lower risk of contracting COVID-19 and hospitalization among the reported cases, although it did not reduce significantly the need for treatment at an intensive care unit or the risk of death [17]. In another meta-analysis, the authors obtained similar results: influenza vaccination did not significantly reduce the risk of hospitalization at an intensive care unit or the risk of death. However, people vaccinated against influenza required mechanical ventilation significantly less often [18].

It is worth emphasizing the importance of seasonal flu vaccinations. Regular, seasonal vaccination also reduces the risk of the establishment of new mutations and thus the risk of a new pandemic.

## 6. Conclusions

On the basis of the analysis of the level of anti-hemagglutinin antibodies in the sera collected from patients in seven age groups in the 2022/2023 epidemic season in Poland, the following conclusions were made:

- The results confirmed the circulation of four strains of influenza viruses in the Polish population: A/Victoria/2570/2019 (H1N1)pdm09, A/Darwin/9/2021 (H3N2), B/Austria/1359417/2021 and B/Phuket/3073/2013. These strains were included in the quadrivalent influenza vaccine formulation recommended by the World Health Organization (WHO) for the 2022/2023 epidemic season for the Northern Hemisphere, including Poland.
- The highest values of geometric mean antibody titres (GMT) and protective rate (%), as compared to other antigens, were recorded for hemagglutinin A/H3N2/.
- Due to the fact that the percentage of the population vaccinated against influenza in the individual age groups was low, the achieved level of protection could be the result of a previous influenza infection.

## Figures and Tables

**Table 1:** Influenza virus strains used for the hemagglutination inhibition assay (HAI) in the 2022/2023 epidemic season.

Epidemic Season 2022/2023		
Influenza virus strains	A/H1N1/pdm09	A/Victoria/2570/2019 (H1N1)pdm09-like virus
	A/H3N2/	A/Darwin/9/2021 (H3N2)-like virus
	B Victoria lineage	B/Austria/1359417/2021 (B/Victoria lineage)-like virus
	B Yamagata lineage	B/Phuket/3073/2013 (B/Yamagata lineage)-like virus

**Table 2:** Determination of the prevalence of antibodies, GMT values and the protective level in two groups: children up to 14 years of age (age groups 1, 2, 3) and the older population (aged 15+; age groups: 4, 5, 6, 7).

Parameter	0–14 Years	15+ Years	Statistical Significance of Difference
Number of subjects	300	400	
A/Victoria/2570/2019 (H1N1)pdm09			
% with antibodies	47.3 [41.7–52.9]%	25.0 [20.8–29.2]%	*p* < 0.001
GMT	39.6 [32.7–48.0]	37.6 [29.8–47.4]	NS
Protection rate	26.0 [21.0–31.0]%	12.3 [9.1–15.5]%	*p* < 0.001
A/Darwin/9/2021 (H3N2)			
% with antibodies	84.3 [80.2–88.4]%	78.3 [74.3–82.3]%	*p* = 0.043
GMT	202.5 [175.8–233.3]	183.1 [160.8–208.6]	NS
Protection rate	79.0 [74.4–83.6]%	74.5 [70.2–78.8]%	NS
B/Austria/1359417/2021 (B/Victoria lineage)			
% with antibodies	19.3 [14.8–23.8]%	23.5 [19.3–27.7]%	NS
GMT	40.5 [30.1–54.5]	32.8 [26.3–40.8]	NS
Protection rate	11.0 [7.5–14.5]%	11.0 [7.9–14.1]%	NS
B/Phuket/3073/2013 (B/Yamagata lineage)			
% with antibodies	54.3 [48.7–59.9]%	69.8 [65.3–74.3]%	*p* < 0.001
GMT	42.3 [36.7–48.7]	30.0 [26.9–33.4]	*p* < 0.001
Protection rate	36.7 [31.2–42.2]%	35.5 [30.8–40.2]%	NS

NS—not significant.

## Data Availability

The raw data supporting the conclusions of this article will be made available by the authors on request.
